# miR-365 Promotes Cutaneous Squamous Cell Carcinoma (CSCC) through Targeting Nuclear Factor I/B (NFIB)

**DOI:** 10.1371/journal.pone.0100620

**Published:** 2014-06-20

**Authors:** Meijuan Zhou, Liang Zhou, Li Zheng, Ling Guo, Yinghui Wang, Hongxia Liu, Chengshan Ou, Zhenhua Ding

**Affiliations:** Department of Radiation Medicine, School of Public Health and Tropic Medicine, Southern Medical University, Guangdong, Guangzhou, People’s Republic of China; East Carolina University, United States of America

## Abstract

Aberrant expression of microRNAs plays vital roles in tumor development and progression. As transcription factors (TFs) are the critical components of signaling cascades, specific targeting effects of microRNAs to specific TFs may determine the role of microRNAs in different cancers. In this study, we identified Nuclear Factor I/B (NFIB) as one of the targets of miR-365 which was previously verified as an onco-miR in cutaneous squamous cell carcinoma (CSCC). Down-regulation of NFIB was a general feature in both CSCC cell lines and tumors from patients which show drastically up-regulated miR-365 expression levels. The siRNA-based knockdown of NFIB mimic the carcinogenic transformation of normal cells by ectopically expression of miR-365 which indicates depletion of NFIB is necessary for miR-365 to exert its pro-carcinogenic function. NFIB may represent a functional barrier targeted by miR-365 to the development of CSCC. Further studies also discovered a conserved feedback regulatory circuitry formed by NFIB and miR-365 in CSCC development which may be potentially utilized as therapeutic target to improve the clinical CSCC treatment.

## Introduction

Cutaneous squamous cell carcinoma (also called cutaneous squamouse cell cancers, CSCC) counts about twenty percent of total skin cancers which is more malignant than basal cell cancers because CSCCs tend to grow and spread much faster than the later. CSCCs commonly develop on sun-exposed areas of the body such as the face, ears, neck, lips, and backs of the hands. Thus Ultraviolet (UV) exposure is a major cause and directly contributes to the occurrence of CSCC.

The current understanding of the molecular mechanism of CSCC is mainly related with transcription factors (TFs), e.g. p53, nuclear factor-kappa B (NF-κB) and activator protein-1 (AP-1) [1]. Mutation or aberrant expression of these TFs contributes directly or indirectly to some or all the cancer hallmarks, including insensitivity to antigrowth or apoptotic signals, self-sufficient growth signals, sustained angiogenesis, limitless replicative potential and invasive or metastatic capability which in turn play important roles in tumorigenesis of CSCC. In addition to causing mutations in the tumor suppressor p53 [2], [3], UVB radiation could induce NF-κB activation and translocation into nucleus which up-regulates IL-6 expression and secretion leading to chronic inflammation. All the above mechanisms will in turn contribute to the development of skin cancer [4], [5].

In addition to the transcriptional events regulated by TFs, the post-transcriptional events in cancer development were recently investigated owing to the fast-growing field of microRNAs. microRNAs are 19–25 nt noncoding RNAs (ncRNAs), which regulate mRNA expression level post-transcriptionally by binding to the 3′ UTR region of target genes. Recent survey of microRNA expression profile in skin cancers led to the identification of a number of aberrantly-expressed microRNAs and their correlation with clinical and pathological features [6]. However, their functions and the following downstream events remain largely unclear [7].

In our previous work, miR-365 was ranked as one of the highest expressed microRNAs induced by UVB treatment and identified as an onco-miR which was highly expressed in both cells and clinical specimens of CSCC [6], [7]. Evidences also supported the role of miR-365 in promoting the development of tumors in nude mice and treatment with antagomiR-365 could inhibit cutaneous tumor formation *in vivo* [6], [7]. The clarified role of miR-365 can help to develop new drug and targeting miR-365-based therapeutic regimen for CSCC. However, the target genes of miR-365 were unclear in CSCC although miR-365 has been shown to targeting cyclin D1, cdc25A, thyroid transcription factor 1 (TTF1) and so on in other cancers [8]–[12]. The lack of detailed molecular mechanism of miR-365 may delay the process of clinical application of anti-miR-365-based therapy in CSCC.

In this work, we performed bioinformatic analysis to find the target gene of miR-365. Evidences from *in vivo* and *in vitro* experiments showed that Nuclear Factor I/B (NFIB) is the target gene of miR-365 in CSCCs. The regulation of NFIB by miR-365 affects the expression of downstream cancer-related effectors, e.g. p53, Bcl-2 and CDK6, which shall be responsible for the development of CSCC.

## Results

### NFIB is Predicted to be the Direct Target of miR-365

As higher expression of miR-365 in CSCC tumors and cells has been verified in the previous study [7], the direct downstream targets of miR-365 become more important for understanding the oncogenic roles performed by miR-365. To gain insights into the miR-365-mediated events in the development of CSCC, we performed target gene screening using web-based algorithms. Among the down-regulated genes, NFIB caught our attention because it was consistently predicted to be a direct target of miR-365 by three well-cited algorithms, TargetScan [13], miRanda [14], miRDB [15]. Moreover, as a known transcription factor, its role in cutaneous carcinogenesis is unclear.

### NFIB is Down-regulated in CSCC Cells and Tumors

**Figure 1:**
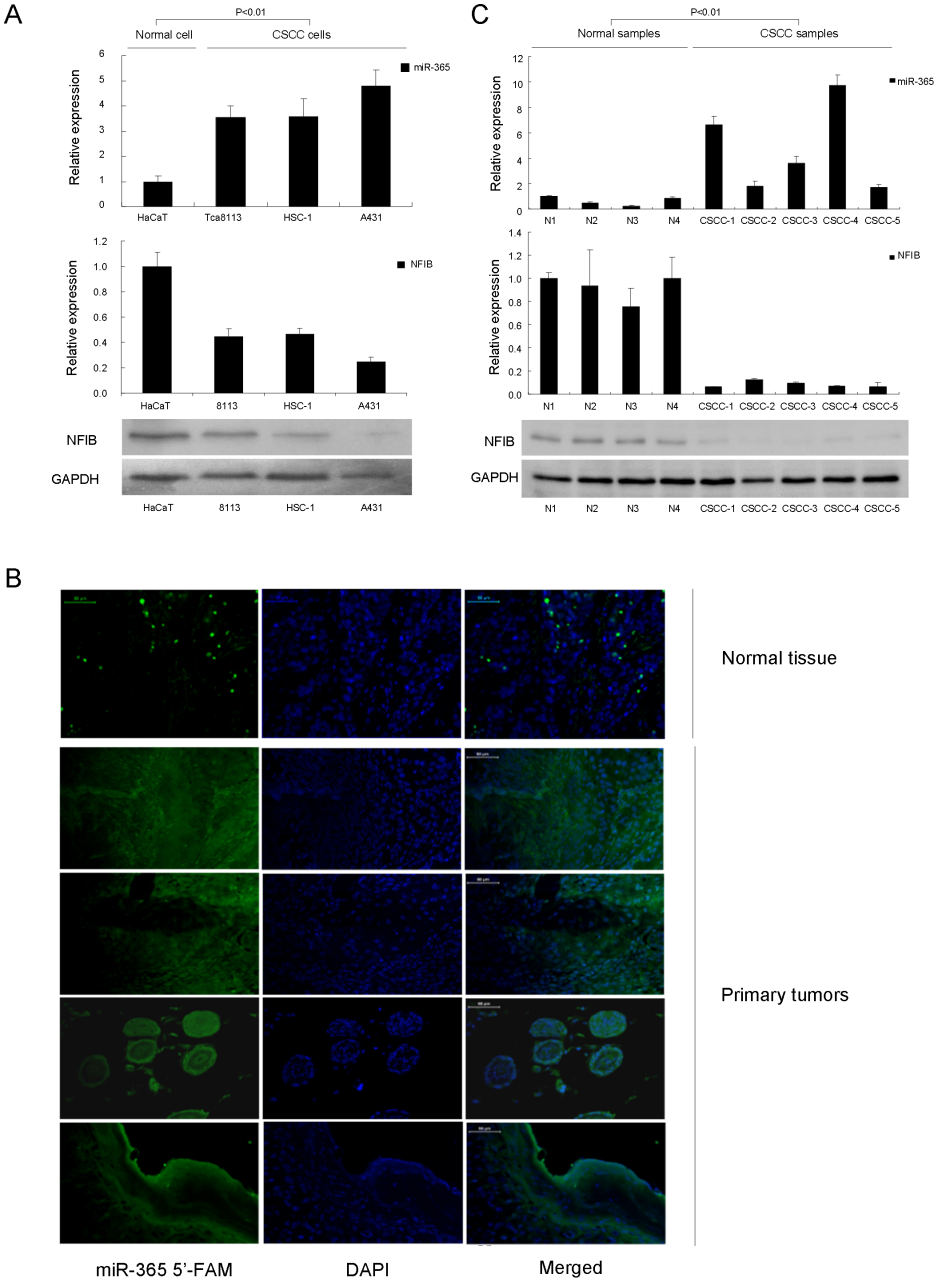
NFIB was down-regulated in CSCC cell lines and primary tumors. (A) The expression of miR-365 and NFIB in CSCC cell lines (Tca8113, HSC-1 and A431) compared with normal HaCaT cells was detected by qRT-PCR and/or western blotting. The expression of NFIB is inversely correlated with miR-365 levels. (B) The expression of miR-365 in CSCC primary tumors was detected by microRNA-FISH using normal skin tissue as control. Bars = 50 µm. (C) Correlation between miR-365 expression and NFIB RNA/protein expression in CSCC primary tumors. The expression of NFIB is inversely correlated with miR-365 levels. In this figure, the expression of miR-365 was examined by qRT-PCR and normalized to U6 snRNA expression. The expression of NFIB protein or mRNA in CSCC tumors compared with normal skin tissue was detected by western blot using as a loading control or qRT-PCR normalized to GAPDH expression. The P value (<0.01) shows significant inverse correlation between the levels of miR-365 and NFIB.

To probe into the possible involvement of NFIB in CSCC development and progression, we began by examining the expression levels of NFIB in CSCC cell lines, A431, HSC-1 and Tca8113, which were compared with normal control (NC), HaCaT cells. Results of western blot showed that the expression of NFIB was down-regulated in all the CSCC cell lines compared with the normal HaCaT cells (Figure 1A). We also checked the miR-365 expression in HaCaT and CSCC cell lines which is inversely correlated with NFIB expression in those cell lines (Figure 1A). To extend this study from cell lines to patient tumors, we first collected and verified miR-365 was overexpressed in clinical patient CSCC tissue samples (Fig. 1B) which is consistent with the previous study [7]. Then NFIB expression in both mRNA and protein levels were then examined in the above tumor samples. As predicted, NFIB expression levels were also inversely correlated with miR-365 in patient tumors (Figure 1C). Collectively, the above results suggest that NFIB is down-regulated in CSCC cell lines and primary tumors in response to the up-regulation of miR-365.

### miR-365 Directly Targets NFIB through Binding to its 3′UTR Region

**Figure 2:**
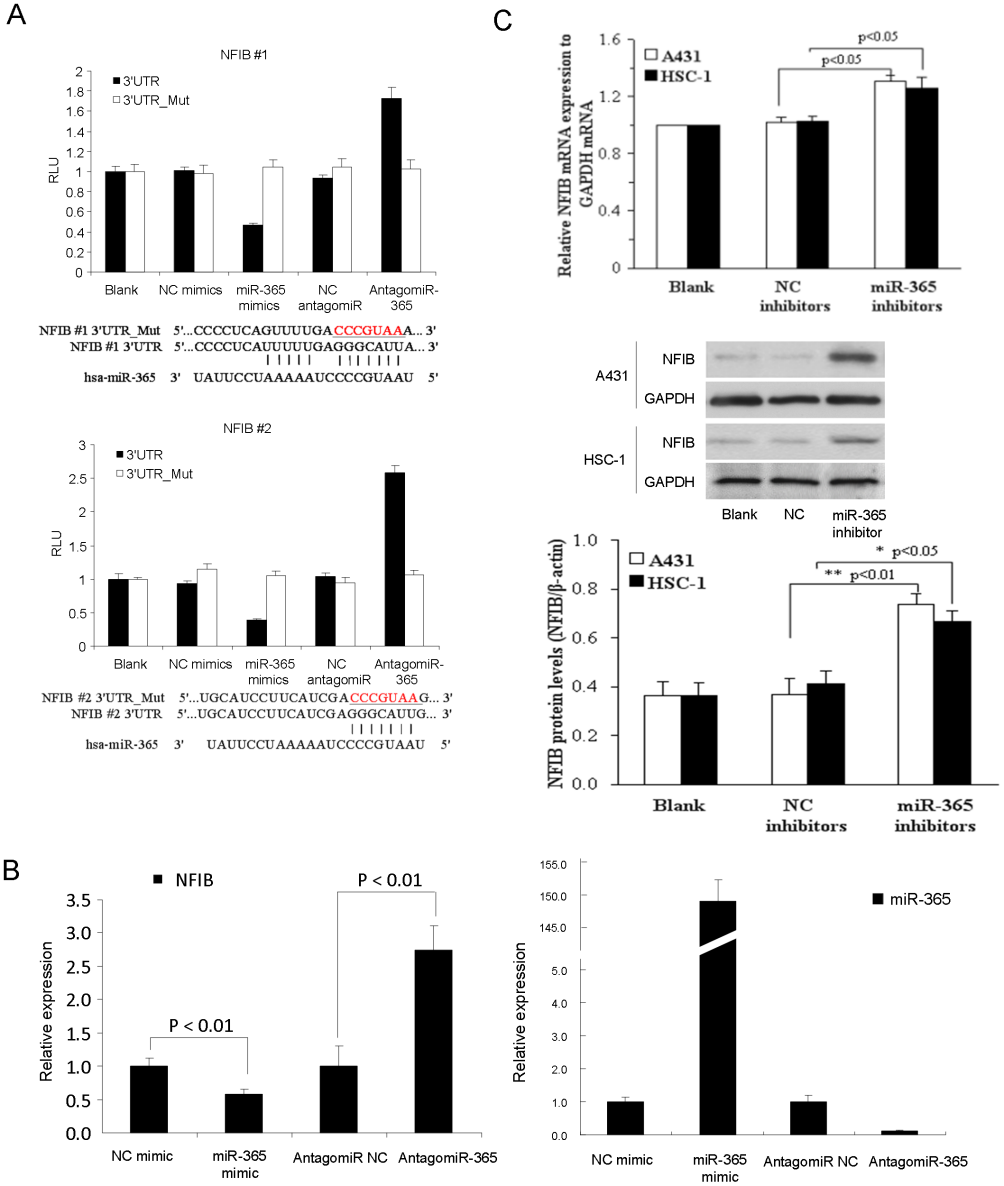
NFIB is targeted by miR-365 in both normal and CSCC cells. (A) Two WT luciferase reporter plasmids were generated by fusing miR-365 binding sites of the NFIB 3′UTR downstream of the luciferase reporter gene. Two mutant plasmids were generated by mutating the binding sites. The mutated sequences were underlined. WT or mutant reporter constructs were then transfected into HaCaT cells with NC or miR-365 mimics. Dual luciferase assay were performed 48 h post transfection and normalized to Renila luciferase activities. Data represent the average of three independent experiments ± SD. (B) NFIB mRNA (Left panel) and miR-365 (Right panel) expression was measured in NC, miR-365, NC inhibitor or miR-365 inhibitor transfected HaCaT cells respectively by qRT-PCR normalized to GAPDH for NFIB or to U6 for miR-365. Expression folds are shown with respect to NC mimic or NC inhibitor transfected cells where normalized copy number was set to 1. (C) qRT-PCR and western blot showing NFIB mRNA and NFIB protein expressed after hsa-miR-365 inhibitors were transferred into A431 and HSC-1 cells. Representative experiments are shown. Means ± SD, n = 4.

To investigate whether the down-regulation of NFIB is due to direct targeting by miR-365, a search for miR-365 binding sites within the NFIB 3′UTR revealed that miR-365 was predicted to hybridize to two evolutionarily conserved sites among vertebrate species (Figure S1A in File S1). Perfect matches exist between the seed regions of miR-365 and the 3′ UTR of NFIB, suggesting that miR-365 can directly repress NFIB expression which were verified by cloning the fragments of the NFIB 3′UTR regions encompassing the target sites to the downstream of the firefly luciferase gene. Cotransfection of miR-365 with each of the two wild-type reporters caused similar repression of the two luciferase reporters (Figure 2A). Such targeting effects were specific to miR-365 binding because the reporter activity was less affected when transfections were repeated with mutant miR-365 binding sites in the NFIB 3′UTR (Figure 2A).

### Inhibition of miR-365 Up-regulates NFIB Expression

To address whether NFIB expression was functionally related with the expression of miR-365 in CSCC cells and tumors, we predicted that miR-365 binding to NFIB 3′UTR would lead to the repression of NFIB. Indeed, ectopic expression of miR-365 by transfection of miR-365 mimic led to decreased expression of NFIB and knocked down of miR-365 by antagomiR-365 could upregulate the expression of NFIB in normal HaCaT cells (Figure 2 B, left panel for NFIB expression and right panel for miR-365 expression). As the downregulation of NFIB in CSCC tumor cells has been shown in Figure 1, we thus treated CSCC cell lines with miR-365 inhibitor and found that NFIB expression was significantly rescued in two different CSCC cell lines, A431 and HSC-1 (Figure 2C). Together, the above results indicated that NFIB is definitely one of the targets of miR-365 in both normal and CSCC cells and knockdown of miR-365 could alleviate the repression and upregulate the expression of NFIB.

### AntagomiR-365-regulated NFIB Expression Inhibits Tumorigenesis *in vitro* and Abolishes the Tumor Growth *in vivo*

**Figure 3:**
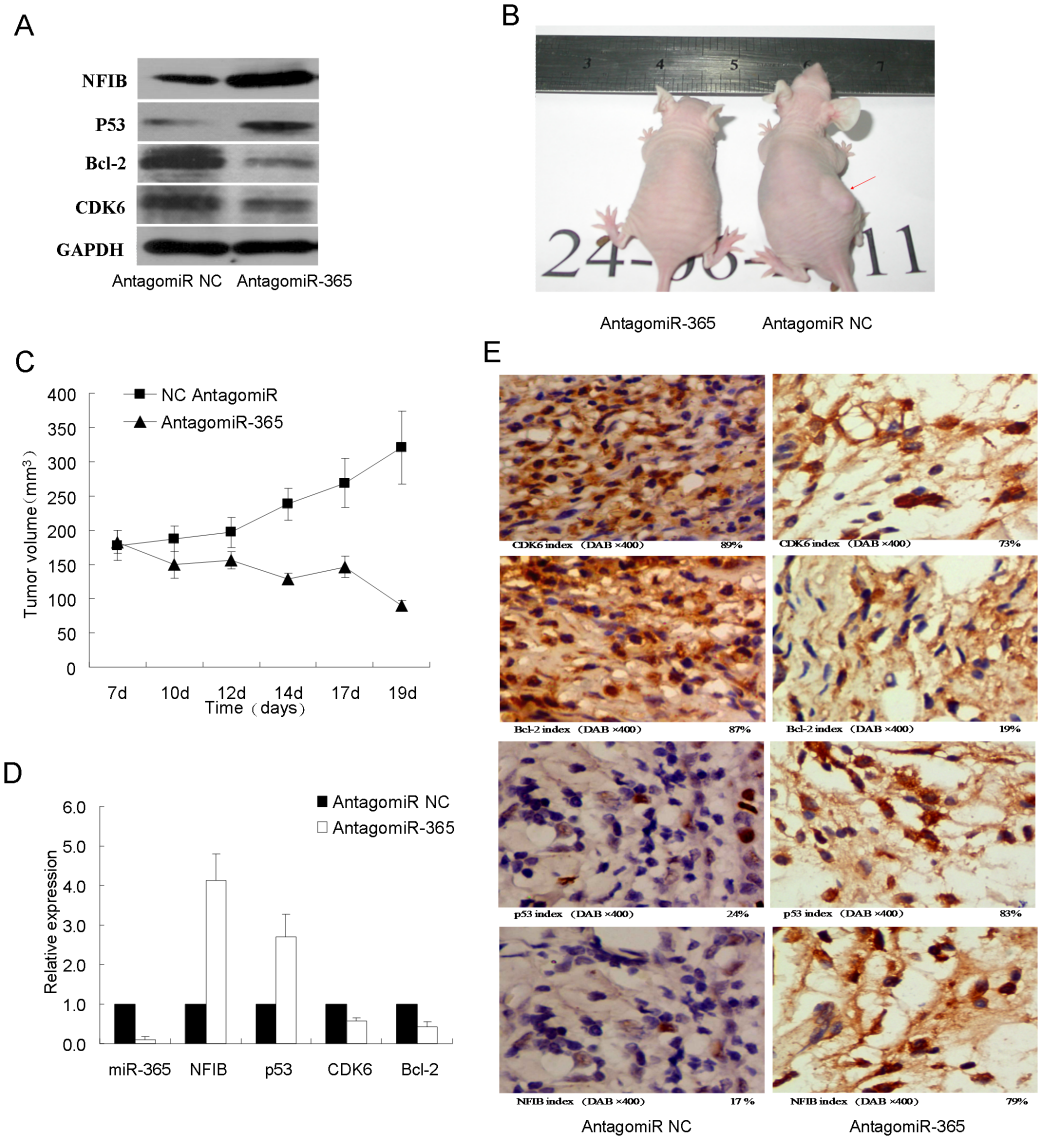
AntagomiR-365 inhibits tumorgenesis and restores NFIB expression both *in vitro* and *in vivo*. (A) The expression of NFIB, p53, CDK6 and Bcl-2 proteins in CSCC cells transfected with antagomiR NC and antagomiR-365 was detected by western blot using GAPDH as a loading control. A representative result is shown here. (B) A representative picture shows the change in tumor volume in xenograft model of BALB/c-nu mice after antagomir-365 treatment 3 weeks with intratumoural injection. The right back flank of BALB/c-nu mice was injected subcutaneously with A431 cells *in vivo* with a volume of more than 150 mm^3^ (n = 5) in comparison with PBS treatment (n = 5). Red arrow head shows the tumor formation from representative mice 21 days after treatment (controls were treated with PBS). (C) Tumor volumes (mm^3^) were recorded in time points as indicated in the growth curve. Relative tumor volumes are shown with respect to day 7. Data are plotted as mean ± S.E. (D) AntagomiR-365 injection drastically decreased the expression levels of the miR-365 in xenografts and thus led to the up-regulation of NFIB and p53 and down-regulation of CDK6 and Bcl-2. Each bar represents the average expression from 5 individual xenografts. Data are plotted as mean ± S.E. (E) IHC staining of NFIB, p53, CDK6 and Bcl-2 on sections of xenograft tumors. Representative fields are shown here and index of positive signal was calculated (n = 10).

To further elucidate the mechanism of NFIB down-regulation in CSCC tumors, we detected the expression of cancer-related genes. p53 is a well known tumor suppressor [16] while Bcl-2 and CDK6 are recognized as pro-carcinogenic factors [17], [18]. Western blot detection in A431 CSCC cells clearly showed that p53 was up-regulated together with NFIB while both Bcl-2 and CDK6 were significantly repressed after treatment of antagomiR-365 (Figure 3A). To firmly reach such conclusion, we thus challenged CSCC tumors formed by injecting A431 cells into nude mice with antagomiR-365 treatment. Tumors growth were greatly suppressed by antagomiR-365 treatment (Figure 3B & 3C) as previous study [7]. We next checked the expression levels of miR-365 and the above mentioned cancer-related genes in tumors. In response to the drastically downregulation of miR-365 in xenografts treated by antagomiR-365, the NFIB and p53 were both upregulated while Bcl-2 and CDK6 decreased (Figure 3D). IHC staining showed that NFIB and p53 were highly up-regulated while Bcl-2 and CDK6 were greatly suppressed in antagomiR-365-treated group than control group (Fig. 3E). Together, the above results might indicate Antagomir-365-regulated NFIB performs tumor-suppressive role in CSCC.

### The Pro-carcinogenic Role of miR-365 is Performed through Targeting NFIB and NFIB can Inversely Regulate the Expression of miR-365 to form a Regulator Circuit

**Figure 4:**
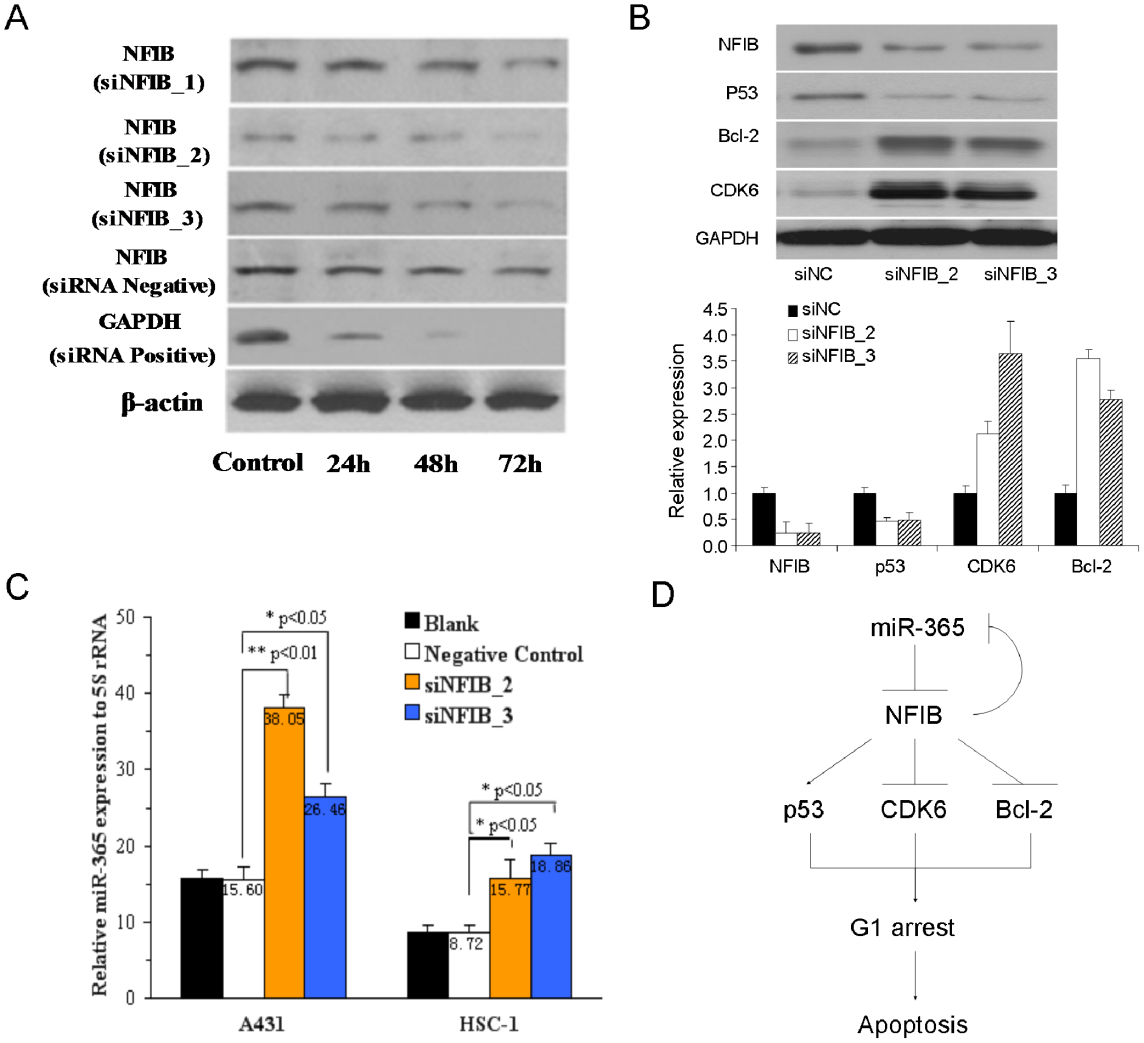
Knockdown of NFIB by siRNA oligos mimics the pro-carcinogenic effects of downregulation of NFIB by miR-365. (A) NFIB protein levels were detected in A431 cells after siRNA oligos of NFIB was transferred and incubated for 24 h, 48 h, 72 h. (B) The expression levels of NFIB, p53, CDK6 and Bcl-2 proteins and mRNA in CSCC cells transfected with two distinct siRNA oligos against NFIB were detected by western blot using GAPDH as a loading control or by qRT-PCR normalized to GAPDH expression. (C) The expression miR-365 examined by qRT-PCR and normalized using U6 snRNA after siRNA NFIB_2, siRNA NFIB_3 and siRNA negative control were transfected into A431 and HSC-1 cells and incubated for 72 h. (D) A mechanism model of miR-365 in CSCC shows that the pro-carcinogenic role of miR-365 is functionally performed through targeting NFIB.

To address whether NFIB plays roles in miR-365 pro-carcinogenic network in CSCC, we knocked down the expression of NFIB by siRNA oligos and checked the downstream responses of carcinogenic regulators. Knockdown assays showed that NFIB can be strongly downregulated by two of the three siRNA oligos targeting NFIB, siNFIB_2 and siNFIB_3 (Figure 4A). Next, the detection of the downstream cancer-related effectors showed that p53 was significantly suppressed while Bcl-2 and CDK6 were highly up-regulated in both RNA and protein levels using the above two effective siRNAs against NFIB (Figure 4B). Such results were consistent with the expression changes of the above carcinogenic regulators after ectopic expression of miR-365 which proved that NFIB is a critical target when miR-365 performs its pro-carcinogenic function.

Many microRNAs can form a feedback regulatory circuit with their targets, e.g. miR-29 with its targets, YY1 and Rybp [19], [20]. To explore this hypothesis, we also detected miR-365 expression after knockdown of NFIB by the above two effective siRNA oligos. As expected, down-regulation of NFIB could strongly up-regulate miR-365 expression (Figure 4C) in two different CSCC cell lines.

## Discussion

In this study, we identified a target gene of miR-365, NFIB, which may represent a barrier to the development of CSCC. Down-regulation of NFIB was a general feature in both CSCC cell lines and tumors from patients which show drastically up-regulated miR-365 expression levels (Figure 1). Knocking down of NFIB by siRNA oligos simulated ectopically expression of miR-365 in normal cells which indicates NFIB is the downstream target of miR-365. Depletion of NFIB is necessary for miR-365 exerting its pro-carcinogenic function.

miR-365 has been identified in colon cancer [11], lung cancer [10], [12], pancreatic cancer [9] and gastric cancer [8]. The role of miR-365 in the development and progression of cancers is debating. While it was regarded as an tumor suppressor in some cancers [8], [11], definite evidences from different studies supported the oncogenic role of miR-365 in many other cancers including skin cancer [7] and pancreatic cancer [9]. The different conclusions might be established due to the differences of dominant molecular mechanism involving the different target gene-mediated signaling pathways in different cancers. The root causes for the development of specific cancers are drastically different. The role of miR-365 in a specific type of cancer is potentially determined by whether the key component of a specific signaling pathway is the target gene of miR-365. In both gastric and colon cancers, miR-365 targeted Cyclin D1 (CCND1) to inhibit cell cycle progression to repress tumorigenesis [8], [11]. However, in pancreatic cancer, miR-365 promote the resistance to Gemcitabine, a standard chemotherapeutic agent for pancreatic cancer, by directly targeted adaptor protein Src Homology 2 Domain Containing 1 (SHC1) and apoptosis-promoting protein BAX [9]. In CSCC, miR-365 was overexpressed in both cells and clinical specimens. Ecotopic expression of miR-365 in normal skin cells could induce subcutaneous tumors *in vivo*. Antagomir-365 treatment inhibited cutaneous tumor formation *in vivo*, along with G1 phase arrest and apoptosis of cancer cells. These results showed that miR-365 acts as an oncogene in CSCC [7]. In breast cancer, miR-365 was identified as one of nine miRNAs that were up-regulated greater than two folds in primary breast cancer compared with normal adjacent tumor tissues (NATs) [21]. These evidences strongly supported the up-regulation of miR-365 is necessary and responsible for those malignancies.

microRNAs exert their functions through target genes, especially transcription factors (TF), e.g. miR-29 which acts as a tumor suppressor by targeting oncogene YY1 [19]. In this study, NFIB is identified as the functional target of miR-365. NFIB is a member of the NFI gene family in vertebrates with versatile transcriptional activities [22], [23]. NFIB functions to regulate more than 100 genes in organs like the brain, lung, liver and intestine [24], and it regulates cell proliferation and differentiation in lung maturation [25]. In human promyelocytic leukemia cell line HL-60, highly up-regulated expression of miR-21 can target NFIB and also NFIB negatively regulated miR-21 expression. Thus NFIB interacts with miR-21 and form a double-negative feedback loop for the survival of leukemia HL-60 [26]. However, NFIB can also act as oncogene in some other cancers. In Small cell lung cancer (SCLC) and triple negative breast cancer, NFIB was highly expressed than normal tissues and repressed apoptosis to promote cell proliferation [27], [28]. The above studies indicate NFIB may perform distinct roles in different cancers.

In this study, originally, NFIB expression is lower in both CSCC cells and patient tumor samples. Down-regulation of NFIB by miR-365 overexpression led to carcinogenic transformation in normal skin cells [7] and enhanced tumorigenesis *in vivo* as demonstrated in this study. Knockdown of NFIB by RNA interference mimics the phenotype and transcriptional responses of carcinogenic regulators which places NFIB as the functional downstream target of miR-365 in miR-365-mediated the pro-carcinogenic pathway. An interesting discovery is that NFIB can inversely regulate miR-365 expression and thus they form a regulatory circuit to manipulate the normal and carcinogenic development of skin cells (Figure 4D), just like miR-29 and YY1 [19].

Here we shall point out that miR-365 may also target other genes, e.g. the above mentioned CCND1. We checked CCND1 expression in CSCC cell lines as well as clinical samples which is also inversely correlated with the higher expression of miR-365 in CSCC cells and tumors (Figure S2A–B in File S1) compared with normal cells and skin samples. And further analysis indicated that CCND1 was negatively regulated by miR-365 (Figure S2C in File S1) like the targeting mechanism in gastric and colon cancers [8], [11]. And knockdown of anti-carcinogenic NFIB in CSCC cells led to the upregulation of CCND1 (Figure S2D in File S1) which is consistence with the enhanced expression of CDK6. The CCND1 and CDK6 can work in the same complex to promote G1 phase progression in cell cycle and contribute to CSCC progression. The discrepancy that the oncogenic CCND1 can be targeted by pro-carcigenic miR-365 which still promotes CSCC progression may due to the multi-targeting nature of miR-365. The NFIB-mediated tumor suppressive pathway may play critical roles in normal skin cells and depletion of NFIB by high level expression of miR-365 in CSCC tumors may disrupted the dominant anti-carcinogenic pathway which contributes the progression of CSCC tumors.

Collectively, our findings discovered NFIB as a novel target of miR-365, which may function in repressing carcinogenic transformation in normal skin tissues. Repression of p53 and upregulation of CDK6 and Bcl-2 by knockdown of NFIB indicates that NFIB may mediate the G1 arrest and the following apoptosis in malignant CSCC cells. Also, our results not only discovered a conserved feedback regulatory circuitry formed by NFIB and miR-365 in CSCC development (Figure 4D), but also showed that this circuitry is potentially utilized as therapeutic target to improve the clinical CSCC treatment.

## Materials and Methods

### Ethics Statement

This study was approved by the Institutional Review Board of Nanfang Hospital affiliated to Southern Medical University, and all patients provided written informed consent for the use of surgical samples. All animals were treated according to standard guidelines for the use and care of laboratory animals.

### Cell Culture and Tumor Samples

CSCC lines A431, Tca8113 (China Center for Type Culture Collection and Cell Bank of the Chinese Academy of Sciences, Shanghai, China) and HSC-1 (Dongguang Biojet Biotech. Co., Ltd, Guangzhou, China) and human benign epidermal keratinocyte cell line HaCaT (China Center for Type Culture Collection, Wuhan, China) were cultured in Dulbecco’s modified Eagle medium (DMEM) supplemented with 10% fetal bovine serum, 100 units/ml penicillin and streptomycin (Invitrogen, Carlsbad, CA) and maintained at 37°C with 5% CO_2_ in a humidified atmosphere. CSCC samples were obtained from patients diagnosed with CSCC from January 2009 to August 2011 in the departments of dermatology, pathology and oncology at Nanfang Hospital and Zhujiang Hospital, affiliated to Southern Medical University and The Third Affiliated Hospital to Sun Yat-sen University.

### Isolation of RNA and Quantitative Real-time-PCR

Total RNAs of the CSCC cell lines (2×10^6^ cells) and tissues (100 mg) were extracted with Trizol (Invitrogen) according to the manufacturer’s protocol. The RNA was quantified by Nanodrop 2000 at OD_260_. The reverse transcription (RT) and quantitative real-time PCR (qRT-PCR) of mRNA were performed with M-MLV 1st Strand Kit (Invitrogen), Oligo(dT)20 primer and SYBR Select Master Mix (Invitrogen). The RT and qRT-PCR of microRNA were performed with TaqMan MicroRNA Reverse Transcription Kit for miR-365 and U6 (Ambion), TaqMan microRNA assay for miR-365 and U6 (Ambion) and TaqMan Universal Master Mix II, no UNG (Ambion). The following primer sequences were used for amplifying each genes listed: NFIB (Forward: TCTCAGCAATGTCAACGAC; Reverse: TTTATGCCTACAGCCTCCT), Bcl-2 (Forward: TCCCTCGCTGCACAAATACTC; Reverse: ACGACCCGATGGCCATAGA), CDK6 (Forward: GAACCAAAATGCCACATACACT; Reverse: TTCGGCCTTTCGCATAGG), GAPDH (Forward: TTGCCATCAATGACCCCTTCA; Reverse: CGCCCCACTTGATTTTGGA). Both of the two types of qPCR reactions were performed on a Stratagene MX3005P instrument. Cycling parameters were 95°C for 10 min, 40 cycles of 95°C (15 s) and annealed/extended at 60°C for 40 s. The gene expression ΔΔCt values of mRNA or miR-365 from each sample were calculated by normalizing with internal control of GADPH or U6 rRNA respectively. Fold change was calculated using the equation 2^−ΔΔCt^. All experiments were performed in triplicates.

### DNA Constructs

To construct the two NFIB-3′ UTR reporter plasmids containing that two miR-365 target sites respectively, two 1000-bp fragments encompassing the two miR-365 binding sites were amplified by two pairs of primers (NFIB-1F: CCGCTCGAGGTTATCTCACCAACGAAGGCTAGG; NFIB-1R: ATAAGAATGCGGCCGCGATTTCAAGGCAAGGCACGTAATG; NFIB-2F: CCGCTCGAGAACATGGACCACTGATTTTGCC; NFIB-2R: ATAAGAATGCGGCCGCGCAAGCAATAACTGACTACTCGTCAC) and cloned into psiCHECK-2 vector (Promega) at the XbaI and NotI sites. Mutant reporter plasmids were generated by mutating the seed region from GGGCAUU to CCCGUAA.

### Oligonucleotides

Precursor microRNA oligo of miR-365 was obtained from Ambion. The 19-nucleotide siRNA duplexes against human NFIB coding region (siNFIB_1: GGCACGAAAGAGAUCAAGA; siNFIB_2: GCACGAAAGAGAUCAAGATAU; siNFIB_3: CCGUGCUGUGUCUUAUCCAAU) and scrambled oligos were obtained from Ribobio. In each case, 50 µM oligos were used for transient transfection into cells with Lipofectamine 2000 (Life Technologies).

### Luciferase Assay

For luciferase experiments, cells were grown in 12-well plates and transfection, including luciferase constructs and oligos, were performed when confluence reaches 70∼80% using Lipofectine 2000 (Life Technologies) according to the manufacture’ manual. 48 hrs after transfection, cell lysates were harvested and luciferase activities were assayed using Dual-Luciferase Reporter Assay System (Promega) and monitored by Varioskan Flash Spectral Scanning Multimode Microplate Reader (Thermo Scientific).

### microRNA-FISH

The formalin-fixed paraffin-embedded CSCC sections were used for miRNA hybridization. Specimen was deparaffinized and hydrated, followed by treatment with proteinase K and refixation in 4% paraformaldehyde. After washing with phosphate-buffered saline and air-drying, the sections were hybridized with the fluorescein isothiocyanate-labeled LNA-miR-365 probe. Nuclei were routinely stained using DAPI.

### Immunoblotting, Immunostaining and Immunohistochemistry

For Western blot (WB) analyses, total cell extracts were prepared and used as previously described [20], [29]. The following dilutions were used for each antibody: NFIB (Abcam; 1∶2000), p53 (CST; 1∶2000), β-Actin (Abcam; 1∶2000), α-Tubulin (Santa Cruz; 1∶2000), Bcl-2 (Sigma; 1∶2,000), CDK6 (CST; 1∶2000), and GAPDH (Santa Cruz Biotechnology; 1∶5000). Densimetric quantification of the Western bands was performed using the Quantity One software (Bio-Rad). Secondary antibodies, including anti-mouse IgG-HRP, anti-rabbit IgG-HRP and anti-goat IgG-HRP, were all purchased from Santa Cruz. The WB results were detected using luminata forte western HRP substrate (Millipore). Immunohistochemistry (IHC) staining on the formalin-fixed paraffin-embedded CSCC sections or xenograft tumor sections was performed using the following antibodies: NFIB (Abcam; 1∶100), p53 (CST; 1∶100), Bcl-2 (Sigma; 1∶100), CDK6 (CST; 1∶100). All the IHC staining sections were captured using Olympus IX51microscope with at least 10 representative images for statistic analysis.

### Treatment of Antagomir-365 *in vitro* and *in vivo*

Antagomirs were synthesized by RiboBio Co. (Guangzhou, China), and the sequences were 5′-APSUPSUPSAPSCGGGGAUUUUUAGGAAUPSAPSChol-3′ (antagomir-365). The A431 cell line was seeded in antibiotic-free media in 6-well plates (2×10^5^ cells/well) and treated for 24 h with antagomir-365 at a final concentration of 100 nM, or with an equal volume of phosphate-buffered saline, when the cells were at 50–60% of confluence. The A431 cell line (2×10^7^ cells) was subcutaneously injected into the right back flank of 5-week-old BALB/c-nu mice. After 1 week, when the tumors reached an average volume of 150 mm^3^, antagomir-365 (25 nM of antagomir-365 diluted in 100 µl phosphate-buffered saline; n = 7), control without antagomir (n = 3), was injected intratumorally three times per week for 2 weeks. Tumor diameters were measured every 2 days.

### Statistical Analysis

Data were presented as mean ± standard deviation (SD). The groups were compared by one-way analysis of variance using SNK-q test or by the t-test with two-tailed P value. Survival data were presented as Kaplan–Meier plots and were analyzed using a log-rank (Mantel–Haenszel) method. The significance level was P<0.05.

## Supporting Information

File S1 Contains the following files: **Figure S1.** miR-365 targets prediction and sequence information. (A) Two of the predicted miR-365 binding sites in the 3′UTR region of NFIB. (B) List of miR-365 targets predicted by TargetScan. (C) Sequence and miRBase ID of human miR-365. **Figure S2.** CCND1 is regulated by miR-365 and NFIB. (A) CCND1 expression in CSCC clinical tumors. (B) CCND1 expression in CSCC cell lines. (C) Human miR-365 targets CCND1 in CSCC cells. (D) Knockdown of siNFIB leads to the upregulation of CCND1 in CSCC cells.
(PDF)
