# Supplementary material for: miR-365 Promotes Cutaneous Squamous Cell Carcinoma (CSCC) through Targeting Nuclear Factor I/B (NFIB)
Source: PLoS One. 2014 Jun 20;9(6):e100620. doi: 10.1371/journal.pone.0100620 (PMC4065106; doi:10.1371/journal.pone.0100620)
Supplement: File S1 — Contains the following files: Figure S1. miR-365 targets prediction and sequence information. (A) Two of the predicted miR-365 binding sites in the 3′UTR region of NFIB. (B) List of miR-365 targets predicted by TargetScan. (C) Sequence and miRBase ID of human miR-365. Figure S2. CCND1 is regulated by miR-365 and NFIB. (A) CCND1 expression in CSCC clinical tumors. (B) CCND1 expression in CSCC cell lines. (C) Human miR-365 targets CCND1 in CSCC cells. (D) Knockdown of siNFIB leads to the upregulation of CCND1 in CSCC cells. (PDF) [file pone.0100620.s001.pdf]

Figure 1S Zhou et al

A

```
Position 1007-1014 of NFIB 3' UTR
5' ...CCCCUCAGUUUUUGAGGGCAUUA...
               ||||| |||||||
3'     UAUUCCUAAAAAUCCCCGUAAU

Position 5572-5578 of NFIB 3' UTR
5' ...UGCAUCCUUCAUCGAGGGCAUUG...
                     |||||||
3'     UAUUCCUAAAAAUCCCCGUAAU
```

B

Target gene	Representative transcript	Gene name	Conserved sites				Poorly conserved sites				Repre-sentative miRNA	Total context+ score	Aggregate PCT	Previous TargetScan publication(s)	Links to sites in UTRs
			total	8mer	7mer-m8	7mer-1A	total	8mer	7mer-m8	7mer-1A					
LPAR5	NM_001142961	lysophosphatidic acid receptor 5	1	1	0	0	2	1	1	0	hsa-miR-365	-0.98	< 0.1	2009	Sites in UTR
SGTB	NM_019072	small glutamine-rich tetratricopeptide repeat (TPR)-containing, beta	1	0	0	1	2	2	0	0	hsa-miR-365	-0.67	0.36		Sites in UTR
TBK1	NM_013254	TANK-binding kinase 1	1	1	0	0	1	0	0	1	hsa-miR-365	-0.65	0.22		Sites in UTR
MAK	NM_001242385	male germ cell-associated kinase	1	1	0	0	1	0	0	1	hsa-miR-365	-0.64	< 0.1		Sites in UTR
NR1D2	NM_001145425	nuclear receptor subfamily 1, group D, member 2	2	2	0	0	0	0	0	0	hsa-miR-365	-0.64	0.67		Sites in UTR
CPT1A	NM_001876	carnitine palmitoyltransferase 1A (liver)	1	0	1	0	2	0	1	1	hsa-miR-365	-0.60	0.24	2009	Sites in UTR
DCUN1D5	NM_032299	DCN1, defective in cullin neddylation 1, domain containing 5 (S. cerevisiae)	1	0	1	0	3	2	1	0	hsa-miR-365	-0.58	0.24	2007	Sites in UTR
ACVR1	NM_001105	activin A receptor, type I	1	1	0	0	1	0	1	0	hsa-miR-365	-0.57	0.64	2007, 2009	Sites in UTR
DLAT	NM_001931	dihydrolipoamide S-acetyltransferase	1	1	0	0	0	0	0	0	hsa-miR-365	-0.53	0.24	2009	Sites in UTR
PIK3R3	NM_001114172	phosphoinositide-3-kinase, regulatory subunit 3 (gamma)	1	0	1	0	1	1	0	0	hsa-miR-365	-0.52	0.24	2009	Sites in UTR
E2F2	NM_004091	E2F transcription factor 2	1	1	0	0	0	0	0	0	hsa-miR-365	-0.52	0.35	2009	Sites in UTR
TRHDE	NM_013381	thyrotropin-releasing hormone degrading enzyme	1	1	0	0	1	0	0	1	hsa-miR-365	-0.50	0.59	2007, 2009	Sites in UTR
RICTOR	NM_152756	RPTOR independent companion of MTOR, complex 2	1	1	0	0	0	0	0	0	hsa-miR-365	-0.49	0.24	2007, 2009	Sites in UTR
GDF11	NM_005811	growth differentiation factor 11	2	1	1	0	1	0	1	0	hsa-miR-365	-0.49	0.30		Sites in UTR
NFIB	NM_001190737	nuclear factor I/B	3	1	1	1	0	0	0	0	hsa-miR-365	-0.49	0.57	2007, 2009	Sites in UTR
RIT1	NM_006912	Ras-like without CAAX 1	1	1	0	0	0	0	0	0	hsa-miR-365	-0.48	0.24	2009	Sites in UTR
RAPGEF4	NM_001100397	Rap guanine nucleotide exchange factor (GEF) 4	1	1	0	0	0	0	0	0	hsa-miR-365	-0.48	0.30	2007, 2009	Sites in UTR
RASSF8	NM_001164746	Ras association (RalGDS/AF-6) domain family (N-terminal) member 8	1	1	0	0	0	0	0	0	hsa-miR-365	-0.48	0.31		Sites in UTR
USP33	NM_015017	ubiquitin specific peptidase 33	1	1	0	0	0	0	0	0	hsa-miR-365	-0.48	0.56	2007, 2009	Sites in UTR
USP48	NM_032236	ubiquitin specific peptidase 48	1	1	0	0	0	0	0	0	hsa-miR-365	-0.48	0.24	2007, 2009	Sites in UTR

C

Human miR-365

hsa-mir-365-1 (MI0000767 HGNC:MIR365-1):

```
acc      aa        ac           ----   -   uuu
   gcaggg  aaugaggg  uuuugggggca    gau gug    c
   ||||||  ||||||||  |||||||||||    ||| |||    c
   cguucu  uuauuccu  aaaauccccgu    cua cac    a
--a      cg        -a           aaua   u   cuu
```

hsa-miR-365a-5p (MIMAT0009199 hsa-miR-365*): 16 - agggacuuuugggggcagaugug – 38

hsa-miR-365a-3p (MIMAT0000710 hsa-miR-365): 56 - uaaugccccuaaaaauccuuau – 77

hsa-mir-365-2 (MI0000769 HGNC:MIR365-2):

```
agaguguucaa g   --       a        ac   c       -----    u   u
           g aca  gcaagaa aaugaggg  uuu aggggca     gcug guu u
           | |||  ||||||| ||||||||  ||| |||||||     |||| |||
           c ugu  cguucuu uuauuccu  aaa uccccgu     ugac cag c
-----gggcua g   ga       g        aa   -       aauac    u   u
```

hsa-miR-365b-5p (MIMAT0022833): 29 - agggacuuucaggggcagcugu – 50

hsa-miR-365b-3p (MIMAT0022834): 68 - uaaugccccuaaaaauccuuau – 89

Figure S1   miR-365 targets prediction and sequence information.

(A) Two of the predicted miR-365 binding sites in the 3'UTR region of NFIB.

(B) List of miR-365 targets predicted by TargetScan.

(C) Sequence and miRBase ID of human miR-365.

Figure 2S Zhou et al

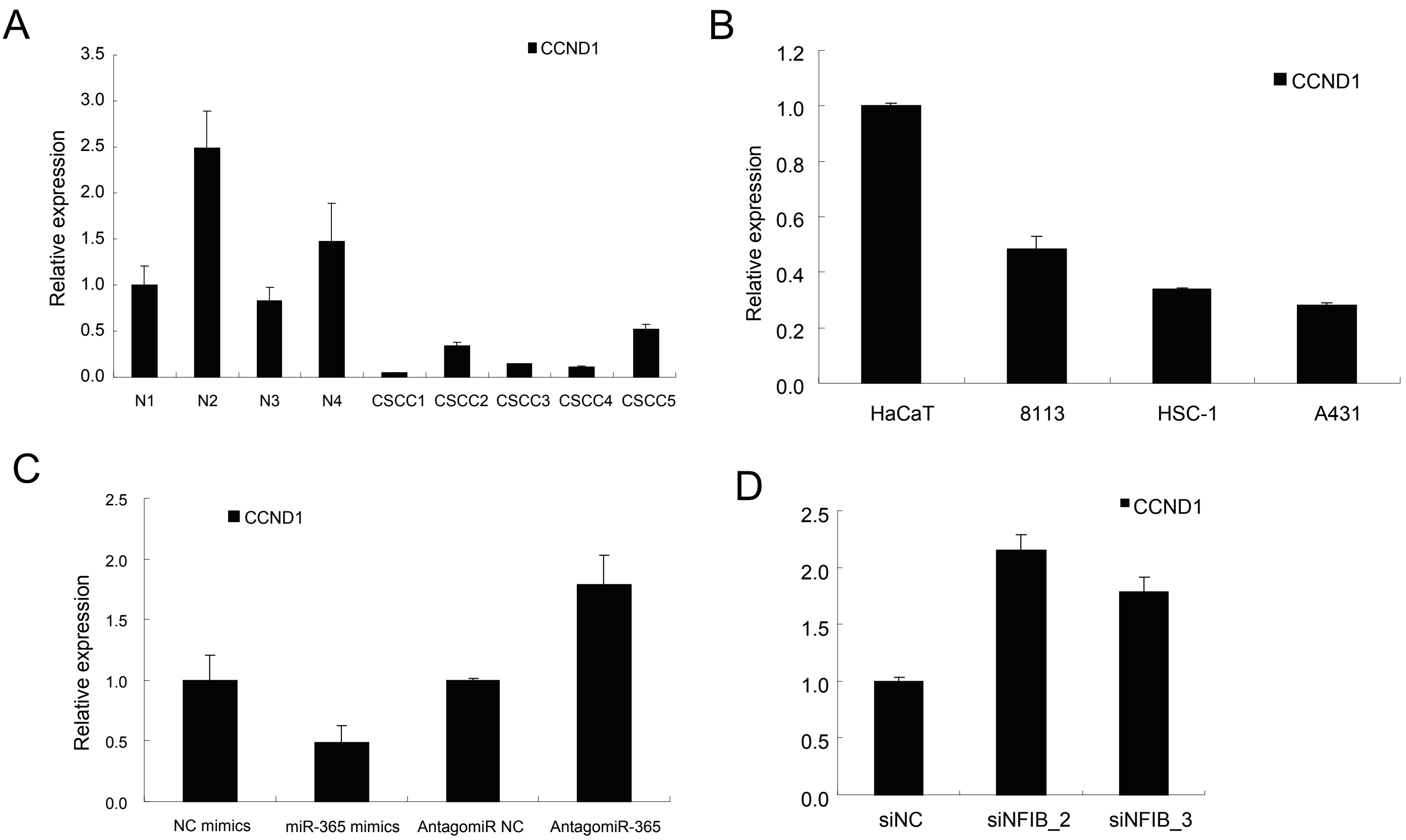

Figure S2 CCND1 is regulated by miR-365 and NFIB

(A) CCND1 expression in CSCC clinical tumors.

(B) CCND1 expression in CSCC cell lines.

(C) Human miR-365 targets CCND1 in CSCC cells.

(D) Knockdown of siNFIB leads to the upregulation of CCND1 in CSCC cells.
